# Transport of N and P in U.S. streams and rivers differs with land use and between dissolved and particulate forms

**DOI:** 10.1002/eap.2130

**Published:** 2020-05-05

**Authors:** David W. P. Manning, Amy D. Rosemond, Jonathan P. Benstead, Phillip M. Bumpers, John S. Kominoski

**Affiliations:** ^1^ Odum School of Ecology University of Georgia Athens Georgia 30602 USA; ^2^ Department of Biological Sciences University of Alabama Tuscaloosa Alabama 35487 USA; ^3^ Department of Biological Sciences Florida International University Miami Florida 33199 USA; ^4^ Department of Biology University of Nebraska at Omaha Omaha Nebraska 68182 USA

**Keywords:** continental scale, ecological stoichiometry, freshwater, NAWQA, nutrient enrichment, watershed

## Abstract

We used a recently published, open‐access data set of U.S. streamwater nitrogen (N) and phosphorus (P) concentrations to test whether watershed land use differentially influences N and P concentrations, including the relative availability of dissolved and particulate nutrient fractions. We tested the hypothesis that N and P concentrations and molar ratios in streams and rivers of the United States reflect differing nutrient inputs from three dominant land‐use types (agricultural, urban and forested). We also tested for differences between dissolved inorganic nutrients and suspended particulate nutrient fractions to infer sources and potential processing mechanisms across spatial and temporal scales. Observed total N and P concentrations often exceeded reported thresholds for structural changes to benthic algae (58, 57% of reported values, respectively), macroinvertebrates (39% for TN and TP), and fish (41, 37%, respectively). The majority of dissolved N and P concentrations exceeded threshold concentrations known to stimulate benthic algal growth (85, 87%, respectively), and organic matter breakdown rates (94, 58%, respectively). Concentrations of both N and P, and total and dissolved N:P ratios, were higher in streams and rivers with more agricultural and urban than forested land cover. The pattern of elevated nutrient concentrations with agricultural and urban land use was weaker for particulate fractions. The % N contained in particles decreased slightly with higher agriculture and urbanization, whereas % P in particles was unrelated to land use. Particulate N:P was relatively constant (interquartile range = 2–7) and independent of variation in DIN:DIP (interquartile range = 22–152). Dissolved, but not particulate, N:P ratios were temporally variable. Constant particulate N:P across steep DIN:DIP gradients in both space and time suggests that the stoichiometry of particulates across U.S. watersheds is most likely controlled either by external or by physicochemical instream factors, rather than by biological processing within streams. Our findings suggest that most U.S. streams and rivers have concentrations of N and P exceeding those considered protective of ecological integrity, retain dissolved N less efficiently than P, which is retained proportionally more in particles, and thus transport and export high N:P streamwater to downstream ecosystems on a continental scale.

## Introduction

Human activities including agriculture, industry and urbanization within watersheds, continue to degrade streams and rivers by dramatically increasing N and P availability (Brown and Froemke 2012, Stets et al. 2020, Wurtsbaugh et al. 2019). Excess N and P enrichment are leading causes of impairment in U.S. streams and rivers, with 41% [N] and 46% [P] of stream miles surveyed by the EPA categorized as ‘poor’ because of nutrient concentrations that exceed ecoregional reference conditions (Herlihy and Sifneos 2008, US EPA 2016). Increased nutrient concentrations modify ecosystem structure and associated processes, including benthic algae and primary production (Van Nieuwenhuyse and Jones 1996, Dodds et al. 2002, Rier and Stevenson 2006), macroinvertebrate communities (Evans‐White et al. 2009, Yuan 2010), and detrital breakdown (Ferreira et al. 2015), driving undesirable effects including blooms of harmful algae, hypoxia, and altered patterns in resource availability and species diversity (Suberkropp et al. 2010, Yuan 2010). Despite the extent of these problems in streams, and their importance for controlling downstream nutrient delivery (Peterson et al. 2001), effects of N and P enrichment on streams and rivers remain understudied compared to those in lakes and coastal ecosystems (Wurtsbaugh et al. 2019). Compared to marine and lake ecosystems, streams and rivers may respond differently to N and P enrichment because of stronger terrestrial linkages (e.g., geology, land use, dependence on terrestrial organic matter; Tank et al. 2010), stronger influence of hydrology (Green and Finlay  2010, Leong et al. 2014), and associated biogeochemical processes that are unique to the unidirectional flow of water (nutrient spiraling; Newbold et al. 1983). Therefore, stream and river biogeochemical processes likely interact in complex ways with point and non‐point nutrient inputs from human activities to affect dissolved and particulate nutrient transport to lakes and coastal zones (Alexander et al. 2008, Dodds and Smith 2016, Bellmore et al. 2018).

Although human activities have increased absolute concentrations of both N and P in streams and rivers, different sources of nutrients diverge in terms of their N and P content (Downing and McCauley 1992); thus, different nutrient sources are likely to shift the balance of N vs. P (i.e., N:P stoichiometry) in receiving waters. For example, high rates of N deposition from transportation, industry, or agriculture, or fertilizer application related to row‐crop agriculture, are likely to increase N more than P (Arbuckle and Downing 2001, Boyer et al. 2002, Fenn et al. 2003). In contrast, nutrient sources such as sewage effluent, runoff from urban watersheds, and amplified erosion of upland soils are likely to increase P relative to N (Downing and McCauley 1992, Withers and Jarvie 2008, Duan et al. 2012). Although the predominant land use within a watershed is expected to affect stream N:P ratios, evidence for such shifts remains less explored in streams and rivers than in lake ecosystems (Downing and McCauley 1992, Vanni et al. 2011, Ginger et al. 2017, but see Maranger et al. 2018, McDowell et al. 2019). Distinct patterns of N and P loading likely prompt shifts in N vs. P limitation of critical ecosystem functions such as nutrient uptake, primary production and detrital breakdown (Rosemond et al., 2002, Tank and Dodds 2003, Dodds and Smith 2016), while also affecting the transport of N, P, and other elements downstream, eventually to coastal ecosystems.

Nutrients in hydrologic transport comprise diverse inorganic and organic forms that can be dissolved or suspended in the water column as particles, dynamically cycling among these different pools as they move downstream (Newbold et al. 1983, Mulholland and Webster 2010). Different nutrient pools are rarely considered in nutrient management frameworks, even though dissolved vs. particulate nutrients differ in terms of their availability for biotic assimilation (dissolved fractions being more available), incorporation into food webs (e.g., filter‐feeding macroinvertebrates; Merritt et al. 2008) and transport downstream (particles being retained more readily; Minshall et al. 2000). Suspended particles are highly complex mixtures of organic (e.g., detritus, autotrophic and heterotrophic microbial biomass, and flocculated aggregates) and inorganic sediments (via rock weathering and soil erosion; Hutchens et al. 2017). Dissolved nutrient concentrations in streams are likely controlled by a combination of point and non‐point source inputs, and the biotic and abiotic transformations that control their uptake and removal from the water column (e.g., microbial assimilation [biotic], denitrification, and adsorption/desorption of P into the particulate pool; Reddy et al. 1999). In contrast, stream particulate nutrient concentrations are likely controlled to a greater degree by upland soil or streambank erosion (Fox et al. 2016) and adsorption/desorption processes, with a more limited role for biotic assimilation and remineralization (Reddy et al. 1999, Uusitalo et al. 2003, Withers and Jarvie 2008).

Differences between dissolved and particulate nutrient sources and processing in streams and rivers likely cause large imbalances between their respective N:P ratios. Suspended particles from upland weathering and erosion processes should be relatively P‐rich compared to dissolved nutrient fractions (Downing and McCauley 1992, Uusitalo et al. 2003). However, suspended matter in streams can also be comprised of autotrophic or heterotrophic biomass (Sakamaki and Richardson 2011): specifically, phytoplankton, bacteria associated with organic matter on particles, or other algal‐ or microbially rich organic particles. If biotic biomass is an important component of suspended particles, then particle N:P ratios may reflect patterns of nutrient assimilation and release that depend on dissolved nutrient availability. Thus, two endpoints in relationships between particulate and dissolved N:P may emerge: (1) particulate N:P changes proportionally with dissolved N:P availability (plasticity/flexibility), indicating Redfield‐like patterns of assimilation and release by biota (Redfield 1958, Sterner and Elser 2002), or (2) particulate N:P is independent of dissolved N:P availability, indicating that particulate N:P ratios are more constant. Such mismatches between dissolved and particulate nutrients are more likely to be driven by abiotic (i.e., chemostatic, *sensu* Godsey et al. 2009), rather than by biotic processes, if predominant contributions of particulates are directly from terrestrial sources, such as streambank erosion (Fox et al. 2016).

We analyzed continental‐scale, publicly available nutrient concentration data (dissolved inorganic and total N and P; hereafter DIN, DIP, TN, TP) from U.S. streams and rivers to address the following questions: (1) How do observed nutrient concentrations compare to documented response thresholds for ecosystem structure and function? (2) How does land use influence concentrations of N and P, and N:P ratios in streams and rivers across large spatial and temporal scales?; (3) Do dissolved and particulate nutrient fractions respond differently to land use or show different temporal trends?; and (4) Do particulate N:P ratios track dissolved inorganic N:P ratios, indicating Redfield‐like patterns of assimilation and nutrient release by stream biota? We hypothesized that a large percentage of observed N and P concentrations would be higher than documented ecosystem structure and function response thresholds for total and dissolved nutrients. Second, we hypothesized that N and P concentrations would increase with human activities associated with agricultural and urban land use, but that agriculture would increase N more than P (higher N:P) and urbanization would increase P more than N (lower N:P). Third, we hypothesized that dissolved N:P ratios would vary intra‐annually and as a function of land use. Last, we hypothesized that if fine particles have a significant microbial component, particulate nutrients would reflect dissolved N:P ratios (e.g., Van Nieuwenhuyse and Jones 1996). Alternatively, we hypothesized that particulate nutrients would not track dissolved N:P ratios, reflecting watershed‐scale drivers that are independent of dissolved nutrient concentrations, and particulate N and P that are relatively inert and driven by abiotic factors. Overall, we expected that N and P concentrations and stoichiometry could inform patterns of nutrient sources related to land use, and so provide insight into processing mechanisms and transport as nutrients move downstream (McDowell et al. 2019).

## Methods

We obtained nutrient concentration data from 2070 sampling sites representing all 50 states as part of the United States Geological Survey (USGS) North American Water Quality Assessment (NAWQA) program (Appendix S1: Fig. S1). Samples were collected between October 1991 and August 2013. Sampling schemes were site‐specific, including a mixture of one‐time sampling events and fixed‐frequency (monthly) sampling. Sampling occurred most frequently during the North American growing season, with ~ 1.6 × more samples taken during the months of March‐September compared to October‐February, on average. We note that the NAWQA sites were specifically selected to capture various anthropogenic impacts; thus, results from this analysis should be interpreted with this design in mind. Similarly, most samples were taken at or near baseflow conditions, whereas high‐flow or storm events were infrequently sampled (~1% of the samples).

We accessed the data using the USGS NAWQA data warehouse (http://water.usgs.gov/nawqa/data, available via the National Water Quality Monitoring Council’s Water Quality Portal: http://www.waterqualitydata.us) and queried available surface water data for ammonia (category includes ammonium ions [NH_4_–N] and un‐ionized ammonia; [Dubrovsky et al. 2010], nitrate plus nitrite (NO_3_–N + NO_2_–N) and orthophosphate (hereafter, dissolved inorganic P, DIP) concentrations in filtered water samples, and TN and TP concentrations from unfiltered, digested water samples.

The number of TN values reported that matched with corresponding dissolved N, dissolved P and TP values from the same location and collection date reduced the final data set in terms of the amount of data (*n* = 7,954), number of represented sites (*n* = 439; Appendix S1: Fig. S1) and sampling dates (September 2003–July 2013). Specific analytical methods and other details for the USGS nutrient data used in this analysis can be found in the Appendix (Appendix S1: Table S1). Dissolved inorganic nitrogen (DIN) concentrations were determined by summing the concentrations of NH_3_–N, NH_4_–N and NO_3_–N + NO_2_–N. Dissolved N:P (i.e., DIN:DIP) and TN:TP were corrected for the molar mass of N and P, unless otherwise noted. We estimated the particulate fraction of N and P (PN or PP) using the difference between total and dissolved inorganic N and P, and corrected for dissolved organic nitrogen (DON) or dissolved organic phosphorus (DOP) (e.g., TN – DIN ‐ DON = particulate N) included in digested TN and TP samples. We calculated the proportion of DON and DOP using data from sites with paired total N and total dissolved N and paired total P and total dissolved P (total dissolved nitrogen [TDN] and total dissolved phosphorus [TDP], *n* = 481, and 12,905 paired records for N and P, respectively; Appendix S1: Fig. S2). We used paired TN/TDN and TP/TDP data because the number of sites with all four variables (TN, TP, TDN, TDP) was limited to 138. Median TN‐TDN was 0.104 mg/L (IQR = 0.04–0.317 mg/L) and median TP‐TDP was 0.037 mg/L (IQR = 0.011–0.112 mg/L). All data organization, analyses, and simulations were performed using R statistical software v.3.4.4 (R Core Team 2019). Total N, P, TDN, and TDP data were retrieved using the ‘*dataRetrieval*’ package in R that retrieves water quality data made available by USGS (De Cicco et al. 2018; see Appendix S1).

We accounted for uncertainty in our estimates of particulate and dissolved organic N and P using bootstrap resampling, whereby we randomly sampled a probability distribution (*n* = 1,000) for the proportion DON and DOP (Appendix S1: Fig. S2) and applied these 1,000 random % DON and % DOP values to each TN‐DIN and TP‐DIP value. We then used a random sample taken from the interquartile range of the resulting bootstrapped distribution of PN or PP values. Thus, particulate N:P was computed with these simulations as (TN–DIN–DON_sim_/TP–DIP–DOP_sim_) and converted to molar ratios. We specifically tested for relationships between % DON and % DOP and land use (see *Land use* below) and found none (simple linear regression, all *P *> 0.05), therefore we moved forward with our bootstrapping approach as described above.

We removed observations where DIN or DIP exceeded TN or TP; these cases represented a small proportion of the DIN (5.2%) and DIP (2.7%) observations in the remaining data set containing matched dissolved N and P and total N and P values. Note, all analyses and results below reflect this smaller data set (*n* = 7,653; 439 sites) and reported analytical methods were the same across all samples for a given analyte. The final data set included dissolved N and P concentrations that occasionally approached analytical limits of detection (see National Environmental Methods Index [https://www.nemi.gov/home/] for analyte‐specific values); data were also reported as “estimated” or “less than” a given value (e.g., <0.001 mg/L). We opted to include these data in our analyses to avoid further left‐censoring the available data (Antweiler 2019). Similar to correcting for DON and DOP, we used Monte Carlo simulations from normal and uniform distributions to ascribe uncertainty to concentration values listed as “estimated” and “less than”, respectively (see Appendix S1).

### Watershed land use

We characterized watershed land use for each station based on the Stream‐Catchment (StreamCat) data set (Hill et al. 2016). StreamCat uses the NHDPlus V2 geospatial framework to summarize landscape attributes of stream segments in the continental U.S. Briefly, we linked GPS coordinates for each NAWQA station provided in the data set to corresponding stream‐segment identifiers (‘COMIDs’) in the StreamCat database using the package ‘*rgdal*’ (Bivand et al. 2019) in R. We then combined segment‐specific watershed‐level land‐use/land‐cover data available in the database (compiled from NLCD 2006), specifically: % crop, % hay, % urban (low, medium, and high intensity), % deciduous forest, % mixed forest, and % coniferous forest. Unless otherwise noted, we combined the separate metrics for agriculture (% hay [i.e., pasture] + % crop;), urbanization (low + medium + high % urban), and forest (% deciduous + % mixed + % coniferous forest; Hill et al. 2017). We converted these percentages to proportions (*x*/100) in our linear models (see *Data analyses* below), treating row‐crop and pasture (i.e., % hay) agriculture separately, to aid comparisons of regression coefficients to previous studies (Arbuckle and Downing 2001, Ginger et al. 2017), and we used percentages in all other aspects of land‐use analyses.

### Data analyses

The first part of our analysis involved comparisons among observed nutrient concentrations and documented response thresholds for ecosystem structure (for total nutrient concentrations) and ecosystem function (for dissolved nutrients). We compared observed TN and TP concentrations to the most conservative (i.e., highest) TN and TP thresholds for benthic algae, macroinvertebrates, and fish communities reported in Evans‐White et al. (2013). We then compared observed DIN and DIP concentrations to reported half‐saturation constants from Michaelis‐Menten/Monod‐type models of algal growth (Schmidt et al. 2019), and leaf litter breakdown rates (e.g., Kominoski et al. 2015). The second part of our analysis was also exploratory and sought to visualize relationships among total, dissolved, and particulate N and P as a function of the proportion of watershed land use characterized as urban, agricultural, or forested. We assessed relationships between land use and total, dissolved and particulate N and P concentrations using locally weighted (loess) regressions across all levels of agricultural and urban land use combined. We used separate loess regressions through the 10th, 25th, 50th, 75th and 90th percentiles of nutrient concentrations to visualize how concentrations changed in terms of both magnitude and variability. We then used a linear modeling approach to quantify differential effects of land use on total, dissolved and particulate N and P concentrations. We used linear mixed‐effects models with log_10_ TN, DIN, or PN, TP, DIP, or PP as the response variable. Models contained the proportion of row‐crop agriculture (pCRO), pasture (pHAY), urban (pURB), or forested (pFOR) land cover in the watershed as predictors, in addition to log_10_ discharge (Q, L/s; Arbuckle and Downing 2001, Ginger et al. 2017). Parameter estimates from the land‐use models described above can be interpreted as the effect of land use on the N or P concentration, where a positive parameter estimate for pAG would indicate higher concentrations of a given nutrient per unit increase in pAG. To directly compare the effects of each predictor, we used standardized coefficients based on scaled predictors (using *z*‐scores). Each model also included a random intercept for monitoring station (*n* = 439) to account for spatial or temporal non‐independence of the data.

To explore potential interactive effects of stream flow rate and land use on N:P ratios, we modeled site‐specific relationships between N:P ratios and discharge with the equation *N:P* = *aQ^b^*, where *N:P* is the nutrient ratio (total, dissolved, or particulate), *Q* is discharge on the date nutrients were sampled, and *b* is the power‐law exponent that describes how N:P ratio changes with discharge (*sensu* Basu et al. 2010). Thus, we generated site‐specific *b* estimates from log‐log slopes of N:P ratio‐Q relationships for a subset of sites with> 10 records of discharge and concentrations of all nutrients (*n* = 94 sites). We then assessed how these *b* coefficients differed across watershed land use. As an example, a site with *b* = 0 indicates static behavior of N:P ratios in response to higher flows. Site‐level daily values of discharge were acquired using the ‘*dataRetrieval*’ package described above.

To test how dissolved and particulate N:P ratios might systematically differ and to infer whether PN:PP ratios were static across DIN:DIP availability, we assessed the relationship between log_10_‐transformed particulate N:P ratio as a function of log_10_ dissolved inorganic N:P ratio to compare the degree of elemental imbalance between particle N:P and dissolved N:P (Schade et al. 2011). In the context of this analysis, we treated particulate N:P as a reflection of ‘ecosystem demand’ and the dissolved inorganic N:P as a reflection of ‘supply’ in the sense of Sterner and Elser (2002), such that the degree of particulate and dissolved elemental imbalance is the parameter *H* in the equation:

(1) $$H\;=\;\text{log}\;\left(c\right)\;+\;\text{log}\;\left(\text{dissolved inorganic N:P}\right)\;\text{/ log}\;\left(\text{particulate N:P}\right)$$

where *c* is a constant, and 1/*H* is equivalent to the slope of the relationship between log_10_ particulate N:P and log_10_ dissolved N:P (Persson et al. 2010). In general, 1/*H* values < 1 would indicate static particle N:P in response to varying dissolved N:P; values approaching zero indicate strictly constant N:P (Sterner and Elser 2002). We co‐opted this stoichiometric model because it effectively quantifies the degree of congruence between dissolved and particulate N:P, providing a potentially useful metric that has implications for the regimes of distinct stream nutrient pools. We recognize that this model is typically used to indicate the degree of elemental homeostasis or plasticity between algae and nutrients, or consumers and their food in optimal growth conditions, and that our data set from streams cannot be used to unequivocally infer the mechanisms driving N:P stasis or non‐stasis; i.e., any association, or lack thereof, between N and P in particulate and dissolved inorganic forms could be mediated by either abiotic or biotic influences or their combination. Regardless of the mechanisms involved, this stoichiometric model effectively quantifies the degree of congruence between dissolved and particulate N:P, providing a potentially useful metric that has implications for the availability and sources of distinct stream nutrient pools. We also tested this model on different subsets of the data, binned by differing absolute concentrations of TP (0.025 vs. 0.1 mg/L), as a proxy for low vs. high autotrophic biomass (Van Nieuwenhuyse and Jones 1996).

We accounted for temporal variability in stream N:P ratios using a subset of streams with >100 samples taken sequentially (for the period September 2003–July 2013). The 13 streams in the subset were located across the contiguous U.S. and generally encompassed the ranges in land use and watershed size we observed across all sites. We compared dissolved and particulate N:P ratios through time for each stream or river, and calculated a stream‐specific *H* value based on the relationship between particulate and dissolved N:P ratios as described above.

## Results

**Fig. 1:**
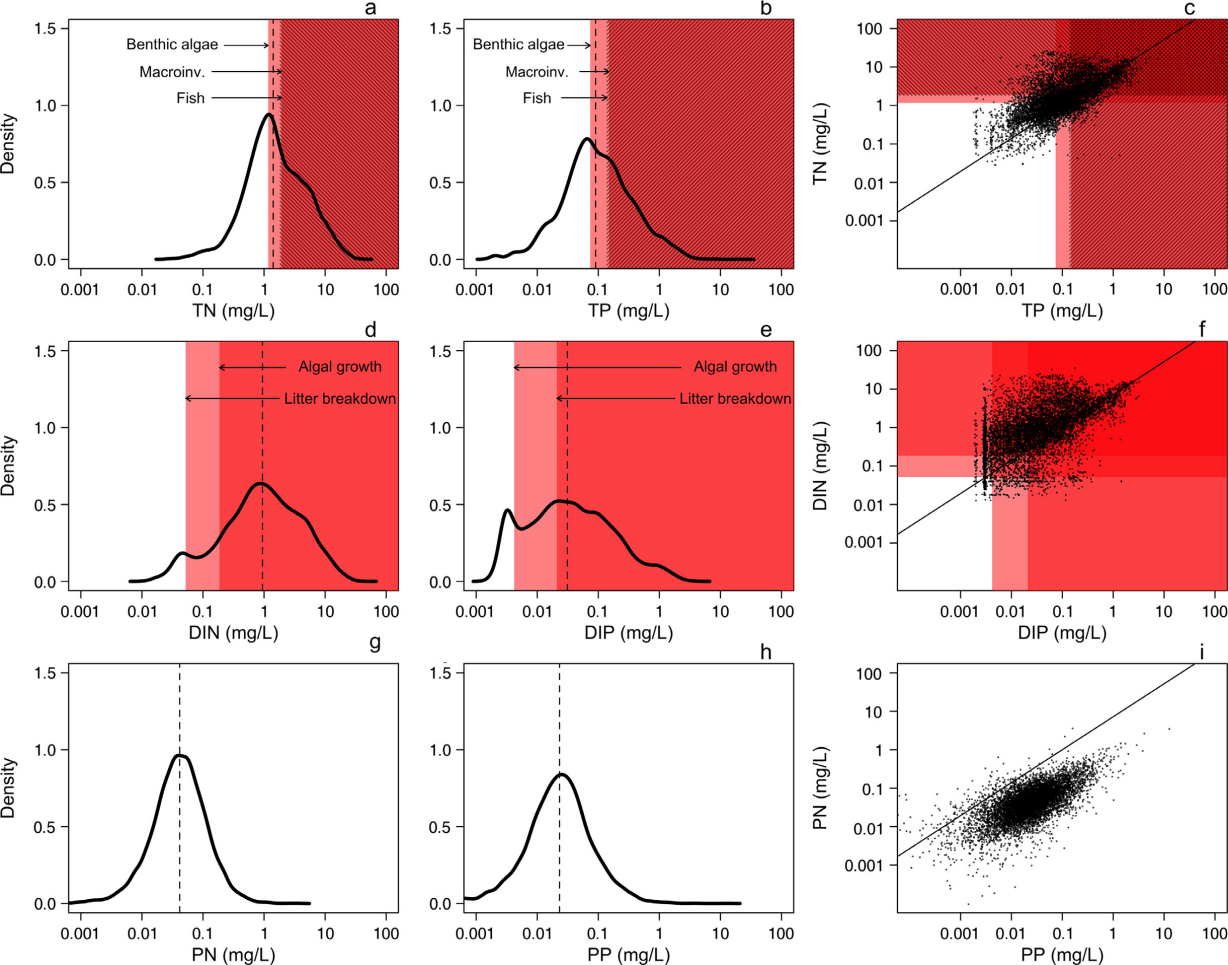
Kernel density plots (generated using Gaussian kernel density estimators at each data point) of log_10_‐transformed total, dissolved, and particulate N and P concentrations (TN [a], TP [b], DIN, [d], DIP [e], PN [g], PP[h]) and bivariate plots of TN vs. TP (c), DIN vs. DIP (f), and PN vs. PP (i) (*n = *7,653 in all cases) from streams and rivers across the United States. We note that the area under each kernel density function is 1, and thus the area under the curve for a given *x*‐axis interval can be interpreted as the probability of observing nutrient values within that range. The solid lines in (c), (f), and (i) indicate the mass ratio of Redfield N:P (7.2). The light red, red, and hashed bands in (a), and (b), (c) indicate the most conservative (i.e., highest) thresholds for structural changes to benthic algae, macroinvertebrates (macroinv.), and fish, respectively, as reported by Evans‐White et al. (2013). The light red, and red bands in (d), (e), and (f) indicate highest reported half‐saturation constants for DIN (d), and DIP (e), for algal growth (Schmidt et al. 2019), and leaf litter breakdown rates (Rosemond et al. 2002, Ferreira et al. 2006, Kominoski et al. 2015), respectively. Vertical dashed lines in (a), (b), (d), (e), (g), and (h), indicate median nutrient concentrations.

Watershed area ranged between 1.2 km^2^ and 220,066 km^2^ (median = 711 km^2^). Mean % agriculture (30%) was higher than mean % forested (25%) and mean % urban (14%) across all watersheds. Median TN and DIN were 1.41 and 0.943 mg/L, respectively; median TP and DIP were 0.091 mg/L and 0.031 mg/L, respectively (Fig. 1). Total N and P were above the highest reported response thresholds for benthic algae in 58% of cases for TN ([threshold] = 1.162 mg/L), and 57% of cases for TP (0.074 mg/L; Fig. 1a, b). Total N and P were above the highest reported response thresholds for macroinvertebrates in 39% of cases (macroinvertebrates; both TN [1.92 mg/L] and TP [0.15 mg/L]), and 40% (TN; 1.83 mg/L) to 37% (TP; 0.139 mg/L) of cases for stream fish (Fig. 1a, b). Dissolved N and P concentrations exceeded highest reported half‐saturation constants for increasing leaf litter breakdown rates in 94% of cases for DIN (0.052 mg/L), and 59% of cases for DIP (0.021 mg/L; Fig. 1d, e), and were above highest half‐saturation constants for algal growth (Schmidt et al. 2019) for 85% of values for DIN (0.186 mg/L) and 87% of values for DIP (0.004 mg/L).

**Fig. 2:**
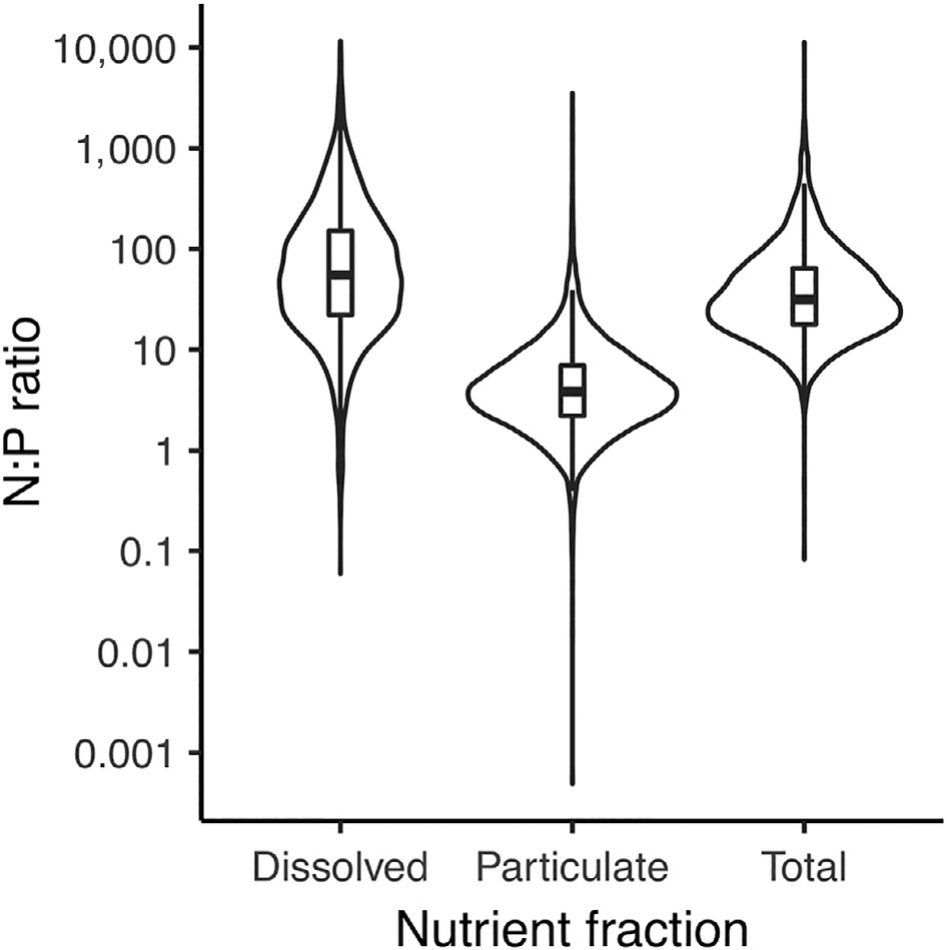
Violin plots of dissolved, particulate, and total N:P ratios. Plots depict the kernel density functions of N:P ratios for each nutrient fraction. Violin plots also contain box plots that show the median (horizontal black line), interquartile range (boxes) and 10–90th percentiles (vertical whiskers) for dissolved, particulate, and total N:P ratios, respectively.

Particulate N:P spanned the smallest range (<1–2,328), followed by total N:P (<1–10,213), and DIN:DIP (<1–11,995). Notably, central tendencies for particulate N:P ratios were below 16:1, with mean N:P ~ 8:1 and median 4:1 (IQR = 2–7; Fig. 2). In contrast, median DIN:DIP (~55:1) was 15.0 × higher than particulate N:P, and median TN:TP (~31:1) was 8.5 × higher than particulate N:P (Fig. 2). Interquartile ranges were also wider for TN:TP (18–64) and DIN:DIP (22–152).

### Watershed land‐use effects on N and P, and N:P ratios

**Fig. 3:**
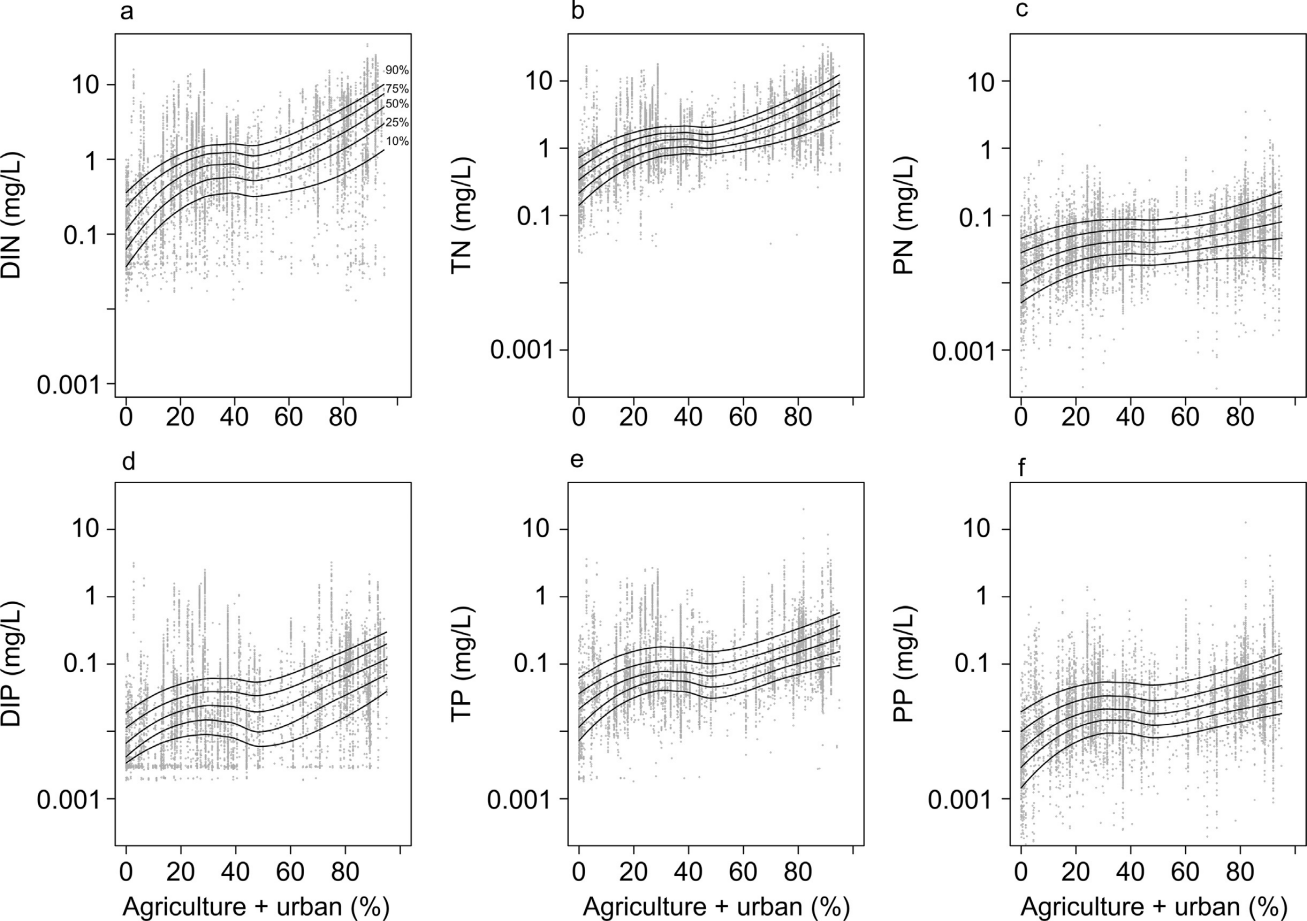
Dissolved (a, d), total (b, e), and particulate (c, f) nitrogen (a–c) and phosphorus (d–f) concentrations as a function of % agricultural (sum of % crop and % hay) plus % urban land use in a given watershed. Each point in gray represents a distinct measurement of N or P concentration at a specific location. The solid lines in (a–f) show locally weighted smoothed (loess) regressions through 10th (lowest line), 25th, 50th, 75th, and 90th (highest line) percentile values of N or P concentration across the sum of watershed‐level % agricultural and % urban land use.

Linear mixed‐effects models relating N or P concentrations to % crop, % hay, % urban, and % forest generally supported trends observed in the locally smoothed plots (Fig. 3). Higher agricultural (considering either % crop or % hay) and urban land use were related to higher total and dissolved N and P concentrations (Table 1). Consistent with our hypothesis, N concentrations tended to increase more with greater row‐crop than urban land use, although TN and DIN concentrations increased as a function of both land‐use types. Agriculture and urban land use had nearly identical effects on TP, and agriculture was associated with higher DIP than urban land use. However, we observed that urbanization had stronger effects on increased P in particles (Table 1). Lower N and P concentrations (all fractions) were consistently associated with higher forested land use (Table 1). In contrast to dissolved and total N and P, relationships between land use and particulate N and P concentrations generally were more muted, reflecting the relatively lower variance in PN:PP ratios, with the exception of particulate P in response to higher discharge, and particulate P in urban watersheds. Discharge had strong, positive effects on concentrations of all forms of N and total and particulate P; standardized effects of discharge were in the same range as those of land use in the models (i.e., >0.1; Table 1).

**Table 1:** Parameter estimates for linear mixed‐effects models predicting N and P concentrations in watersheds of the continental U.S.

Model	Intercept	Q	pCRO	pHAY	pFOR	pURB	Marg‐*R* ^2^	Cond‐*R* ^2^
Log_10_ TN	−**0.387**	**0.114 (0.142)**	**0.796 (0.240)**	**0.774 (0.099)**	−**0.741 (−0.161)**	**0.523 (0.120)**	0.451	0.849
Log_10_ DIN	−**1.092**	**0.162 (0.203)**	**1.183 (0.357)**	**1.205 (0.154)**	−**0.469 (−0.102)**	**1.025 (0.235)**	0.345	0.799
Log_10_ PN	−**1.561**	**0.088 (0.110)**	**0.226 (0.068)**	0.060 (0.008)	−**0.979 (−0.213)**	0.111 (0.025)	0.293	0.572
Log_10_ TP	−**1.344**	**0.115 (0.143)**	**0.283 (0.086)**	0.286 (0.037)	−**1.161 (−0.253)**	**0.386 (0.088)**	0.412	0.777
Log_10_ DIP	−**1.515**	0.003 (0.004)	**0.399 (0.121)**	0.346 (0.044)	−**0.983 (−0.214)**	0.332 (0.076)	0.202	0.747
Log_10_ PP	−**1.647**	**0.169 (0.280)**	0.135 (0.067)	0..211 (0.024)	−**0.759 (−0.271)**	**0.324 (0.097)**	0.373	0.697

Notes

Each model included parameters for the proportion of each land‐use type in the watershed (pCRO, pHAY, pFOR, pURB), and log_10_ discharge (Q L/s) and a site‐level random intercept. The effect of pCRO, pHAY, pFOR and pURB can be interpreted as the effect on N or P concentration when log_10 _(Q), and land‐use types are held constant at their mean. The standardized effect (standardized predictors using *z*‐scores) of each predictor is also shown in parentheses next to the corresponding parameter estimate to allow direct comparisons among predictors. Model fits (marginal and conditional‐*R* ^2^) are also presented. Bold text emphasizes parameter estimates with significance probabilities < 0.05 and 95% confidence intervals that exclude zero.

Nutrient concentrations increased as a function of agriculture and urban land use together. Locally smoothed regression plots showed increasing N and P concentrations with higher % agriculture (sum of % crop, % hay) plus % urban land use, especially between low to moderate levels (10–40%) of agricultural plus urban land cover (Fig. 3a–f). These patterns were strongest for dissolved and total N and P; particulate N and P showed positive, but weaker, relationships with % agriculture + % urban land use. Land use was generally a poor predictor of the percentage of particle and dissolved nutrients (as a fraction of total N or P). Percent particulate N declined slightly with % crop and % urban land use (linear mixed‐effects model indicated 0.02% decrease for a 1% increase in either crop or urban land use; *P < *0.001 and *P < *0.017, respectively), whereas % particulate P and % dissolved N and P were unrelated to % crop or % urban land use (all *P > *0.05).

There was substantial variability in nutrient concentrations regardless of land use; however, the upper and lower bounds of nutrient concentrations encompassed increasing values with greater % agriculture + % urban land use (Fig. 3a–f). For example, the interquartile range of DIN was 1.2–9.2 mg/L when developed land use was between 85–95% (*n* = 993; Appendix S1: Table S2); these values are 12.4 × and 12.5 × the lower and upper bounds of the IQR, respectively, when developed land was between 5–15% (*n* = 676; IQR DIN developed land 5–15% = 0.10–0.74 mg/L; Appendix S1: Table S2).

**Fig. 4:**
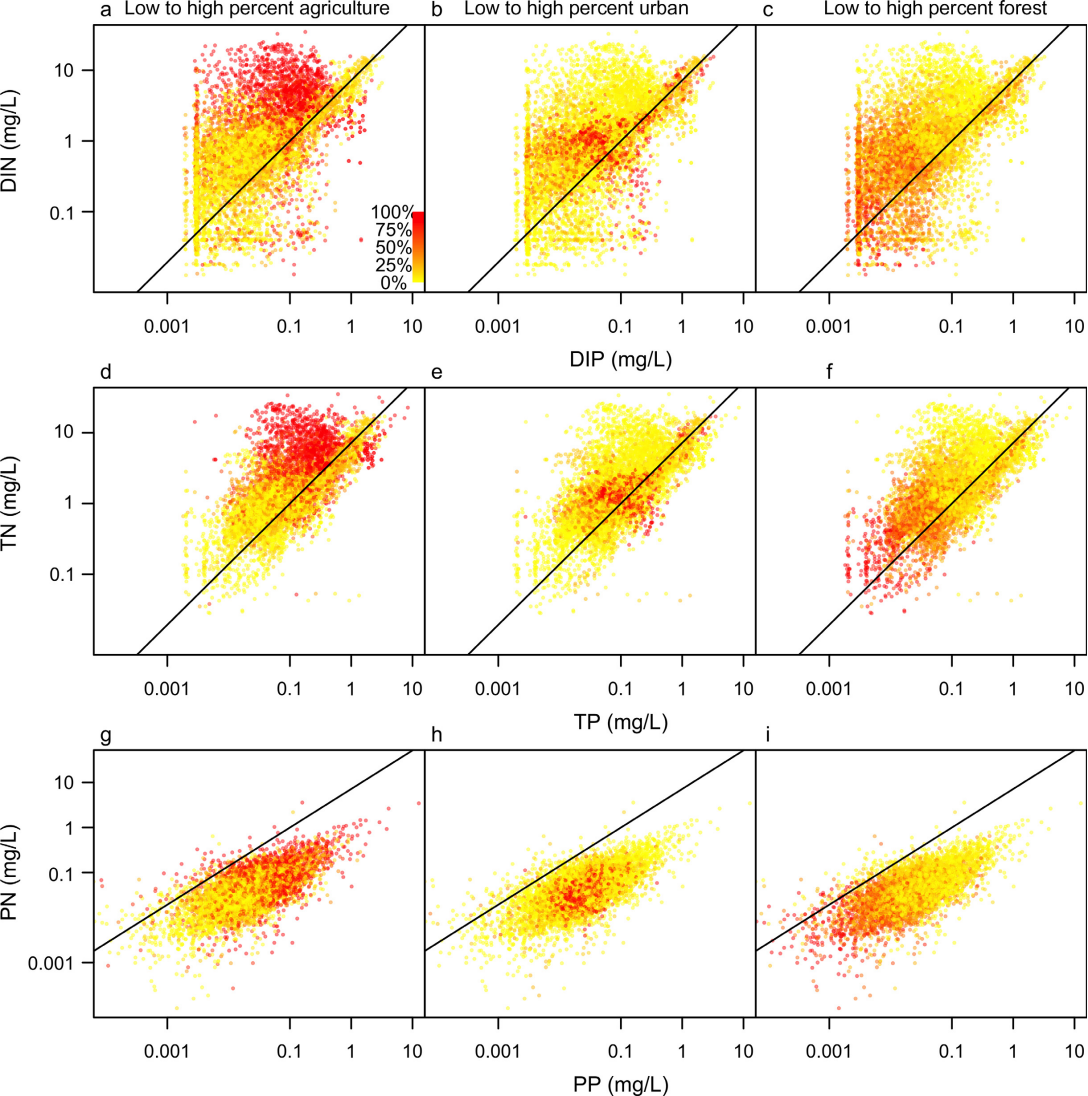
Dissolved inorganic (a‐c; DIN, DIP), total (d‐f; TN, TP) and particulate (g‐i; PN, PP) nitrogen vs. phosphorus (mg/L) in streams and rivers of the United States. Each point is color‐coded to transition from yellow to red to indicate the proportion of the sampling‐location watershed with agricultural (a, d, g), urban (b, e, h), or forested (c, f, i) land use; redder colors indicate higher proportions of each land‐use type. The solid lines in each indicate an N:P ratio (by mass) of 7.2 (Redfield N:P). Note that *y*‐axis scales are slightly different in (g–i).

We detected different relationships between TN:TP and DIN:DIP as a function of land‐cover metrics, based on inspection of plots relating total, dissolved, or particulate N vs. P (Fig. 4a–i). Dissolved N:P ratios were more variable than TN:TP ratios, but we observed similar visual patterns in the location of high N concentrations within N vs. P plots. For example, DIN:DIP ratios tended to occur above 16:1 in highly agricultural catchments (Fig. 4a), whereas DIN:DIP for urban and forested watersheds were more closely aligned with the Redfield N:P line (Fig. 4b,c). Similarly, TN:TP ratios in highly agricultural watersheds tended to occur above the Redfield ratio (Fig. 4d), whereas TN:TP ratios in urban or forested watersheds were more closely aligned with N:P ratios approaching Redfield N:P (Fig. 4e,f).

**Fig. 5:**
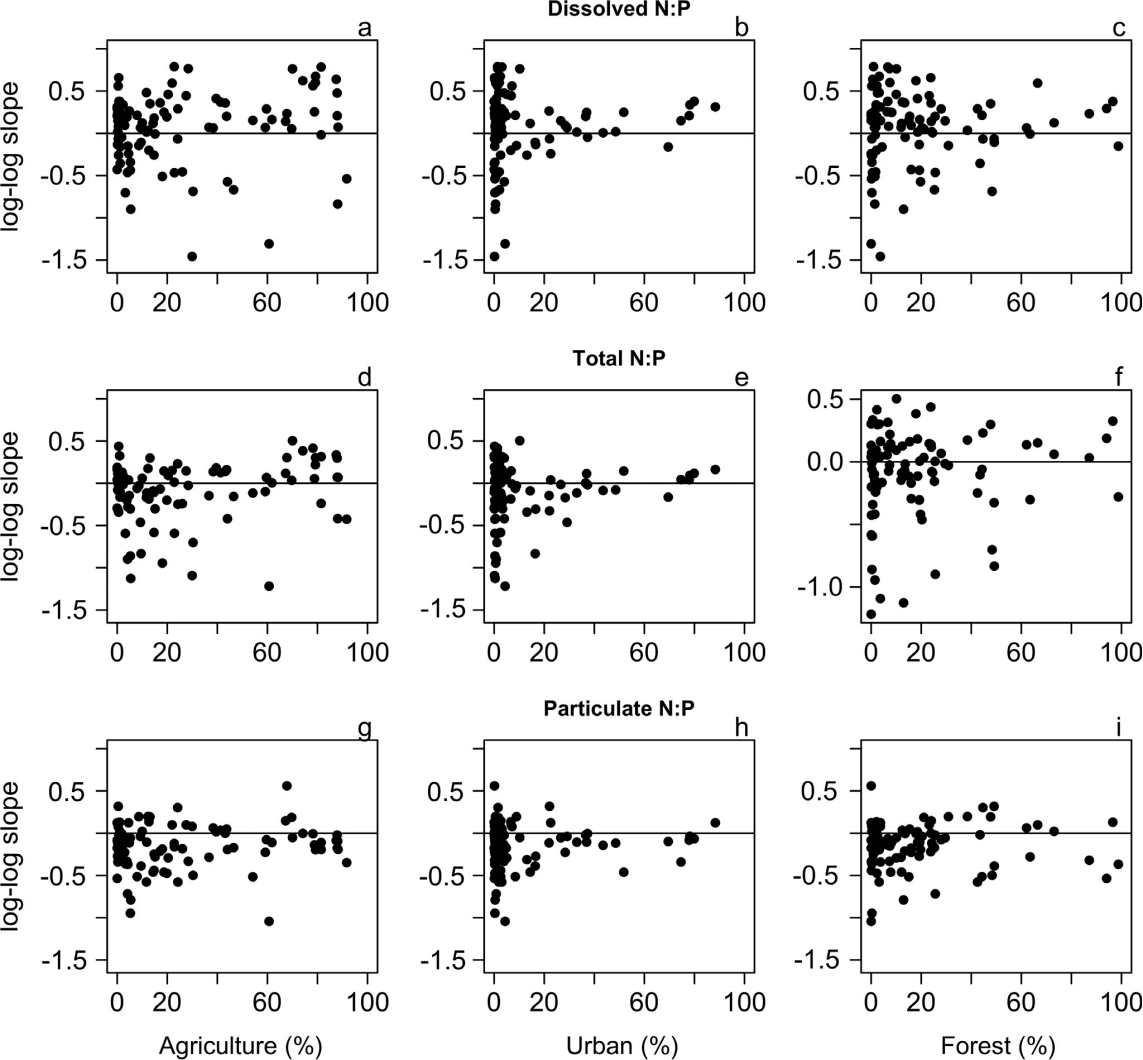
Site‐specific (*n* = 94) discharge‐N:P ratio log‐log coefficients, plotted as a function of % agriculture (a, d, g), % urban (b, e, g), or % forest (c, f, i). Coefficients for dissolved (a–c), total (d–f) and particulate N:P (g–i) vs. discharge are shown. Coefficients increased for total N:P; (*P* = 0.03) as a function of agricultural land use (d). Horizontal solid lines indicate a Q vs. N:P log‐log coefficient = 0.

Stream flow rate affected stream N:P ratios, as evidenced by discharge‐N:P ratio slopes (Fig. 5a–i). On average, dissolved N:P ratios were more likely to increase with discharge (62 of 94 sites), whereas total N:P ratios increased in 42 of 94 sites, and the majority of sites (73 of 94) showed decreasing particulate N:P with higher discharge. We found limited evidence for interactive effects of land use and discharge on N:P ratios. In agricultural watersheds, discharge‐N:P ratio slopes were significantly higher for total N:P (simple linear regression; *P* = 0.03; Fig. 5d), but otherwise coefficients were largely unrelated to watershed land use.

### Relationships between dissolved and particulate nutrient pools

**Fig. 6:**
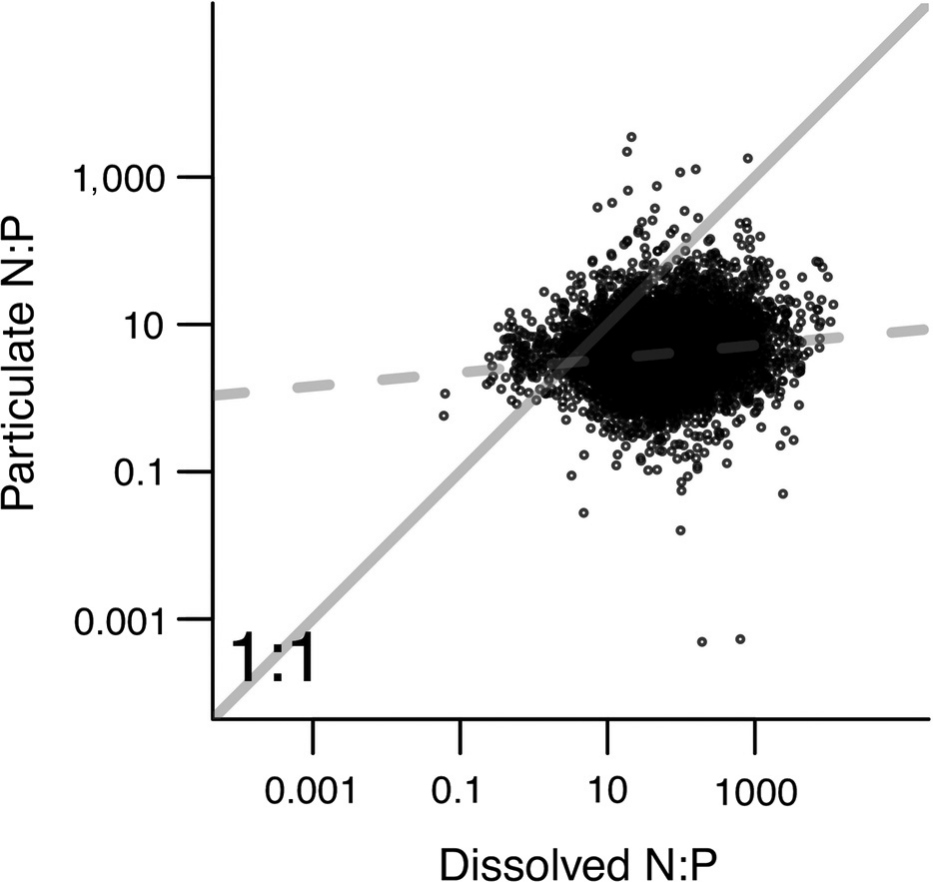
Particulate N:P vs. dissolved inorganic N:P ratios in streams and rivers of the United States. The solid gray line indicates a 1:1 relationship, and the dotted line shows the particulate N:P vs. dissolved N:P relationship (log_10_ particulate N:P = 0.434 + 0.096 * log_10_ dissolved N:P).

Particulate and dissolved N:P were both variable and spanned several orders of magnitude, respectively (Fig. 6). However, compared to dissolved N:P ratios, for which 50% of N:P values were in the range of 22–151, 50% of particulate N:P values fell within the range of 2–7 (IQR). Accordingly, particulate N:P ratios were relatively static as a function of wide‐ranging DIN:DIP ratios (Fig. 6). Consistent with this visual trend, we found a 1/*H* value that was suggestive of constant particle N:P (i.e., 1/*H* = 0.096; *P* << 0.001; Sterner and Elser 2002, Persson et al. 2010) across dynamic dissolved N:P. We also tested for changes in the 1/*H* value with increased TP concentrations (low [0.025 mg/L] vs. high [0.10 mg/L) as a proxy for higher prevalence of suspended autotrophic biomass (Van Nieuwenhuyse and Jones 1996, Wurtsbaugh et al. 2019). The 1/*H* value for particulate and dissolved N:P was slightly negative when TP was either low (TP < 0.025 mg/L) or high, but especially when TP exceeded 0.10 mg/L (1/*H =* −0.023, *P = *0.36 when TP < 0.025 mg/L; 1/*H* = −0.036, *P* < 0.001 when TP> 0.10 mg/L).

### Temporal variability in dissolved and particulate N:P

**Fig. 7:**
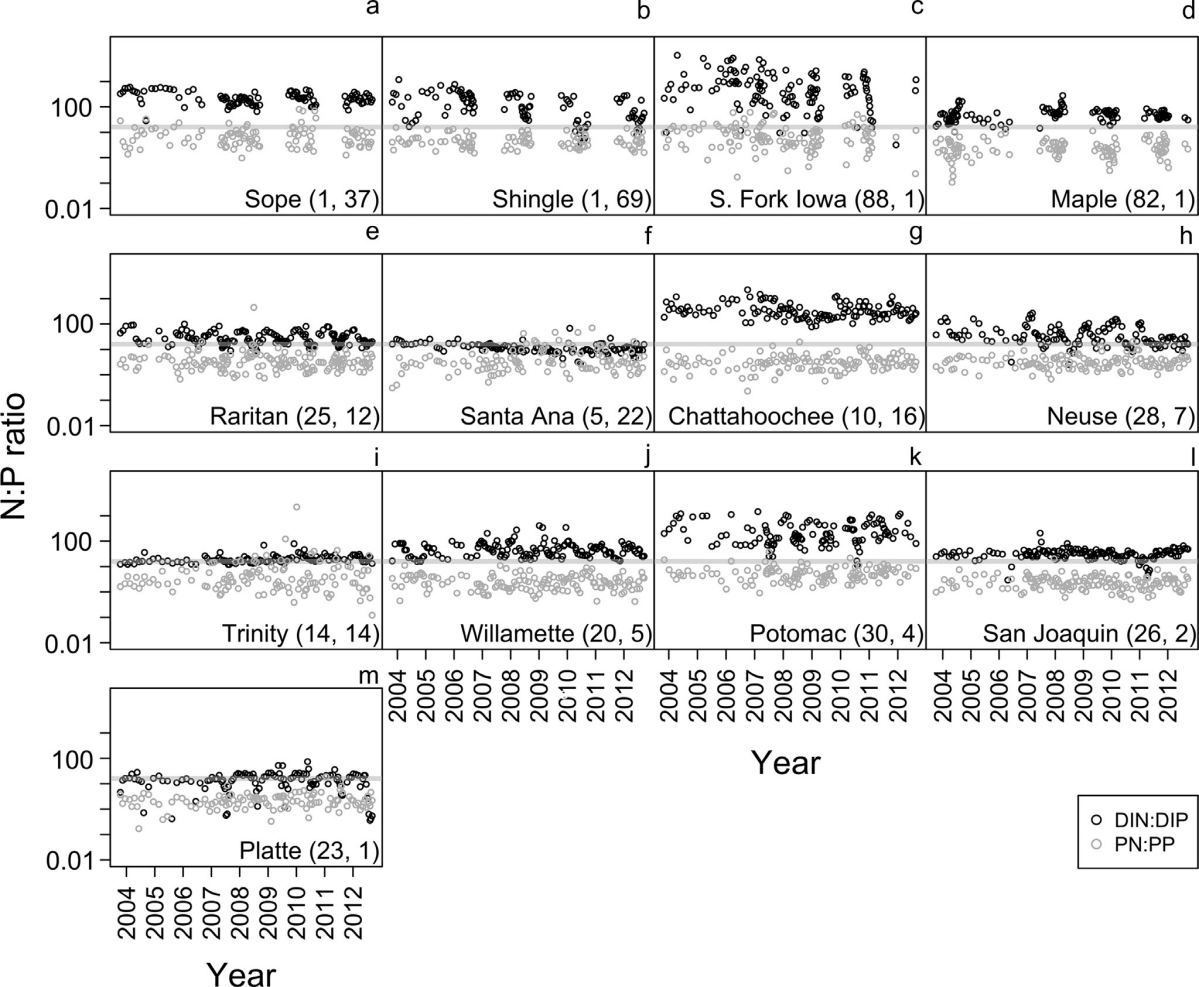
Temporal patterns of dissolved N:P (black circles) and particulate N:P (gray circles) in 13 rivers representing distinct land uses and watershed sizes. Numbers in parentheses after each stream or river name indicate corresponding % agriculture and % urban land cover, respectively. Note that *y*‐axes are presented on a log_10_ scale. The solid horizontal gray line indicates Redfield N:P (16:1) as a point of reference. All ratios are molar.

The subset of 13 streams with >100 sequential samples spanned similar gradients to the larger set of streams in terms of watershed size (86–210,860 km^2^), land use (% urban = 0.7–69.6; % forested = 1.7–58.6; % agriculture = 0.6–88.2), and N:P ratios (range of median DIN:DIP for each stream = 11–505; Table 2). Similar to overall trends, dissolved N:P ratios were highly variable among the 13 streams, whereas particulate N:P was less variable during the time‐series presented here (Table 2, Fig. 7a–m).

**Table 2:** Location (latitude and longitude), watershed area (km^2^), land use (%), and medians [interquartile range] for total (TN:TP), dissolved (DIN:DIP) and particulate N:P (PN:PP) ratios in the subset of 13 streams with >100 sequential samples.

Name	Lat.	Long.	WSA	URB	FOR	AG	DIN:DIP		PN:PP		TN:TP		1/*H*	*P*‐value
Sope	33.95	‐84.44	86	36.6	22.1	0.6	220	[146‐327]	6	[4‐11]	85	[57‐127]	‐0.443	0.002
Shingle	45.05	‐93.31	103	69.6	4.4	1.1	98	[33‐262]	4	[3‐6]	26	[20‐37]	‐0.029	0.480
S. Fork Iowa	42.32	‐93.15	583	0.7	1.9	88.2	505	[159‐1308]	7	[3‐13]	211	[81‐466]	0.287	<0.001
Maple	41.56	‐96.54	962	0.8	1.7	81.5	48	[35‐65]	3	[1‐5]	30	[19‐40]	0.194	0.198
Raritan	40.56	‐74.53	2072	12.2	38.5	24.6	22	[17‐39]	3	[2‐4]	20	[16‐29]	‐0.349	0.002
Santa Ana	33.88	‐117.65	5681	21.9	11.6	4.8	11	[9‐15]	3	[2‐6]	11	[9‐13]	‐0.792	<0.001
Chatta‐hoochee	33.48	‐84.90	6252	16.4	49.4	9.5	301	[209‐492]	3	[2‐4]	85	[53‐117]	0.120	0.207
Neuse	35.26	‐77.59	7020	6.7	33.9	27.5	29	[18‐58]	3	[2‐4]	18	[15‐22]	‐0.030	0.624
Trinity	32.71	‐96.74	16253	14.4	12.6	14.4	18	[15‐23]	3	[2‐6]	16	[14‐19]	‐1.074	0.003
Willamette	45.52	‐122.67	28922	5.0	53.9	20.4	45	[27‐69]	3	[1‐4]	24	[19‐30]	‐0.331	<0.001
Potomac	38.93	‐77.12	29967	3.8	58.6	29.6	187	[101‐456]	6	[3‐8]	59	[43‐88]	0.015	0.824
San Joaquin	37.68	‐121.27	35855	2.0	28.3	26.0	32	[24‐40]	2	[1.5‐3.1]	22	[16‐24]	0.046	0.635
Platte	41.02	‐96.16	210860	1.3	8.0	22.8	15	[9‐21]	2	[1 ‐ 3]	11	[9‐14]	‐0.063	0.308

Note

Also shown are the 1/*H* values for the particulate vs. dissolved N:P relationships for each stream, with associated *P‐*values of these slopes.

**Fig. 8:**
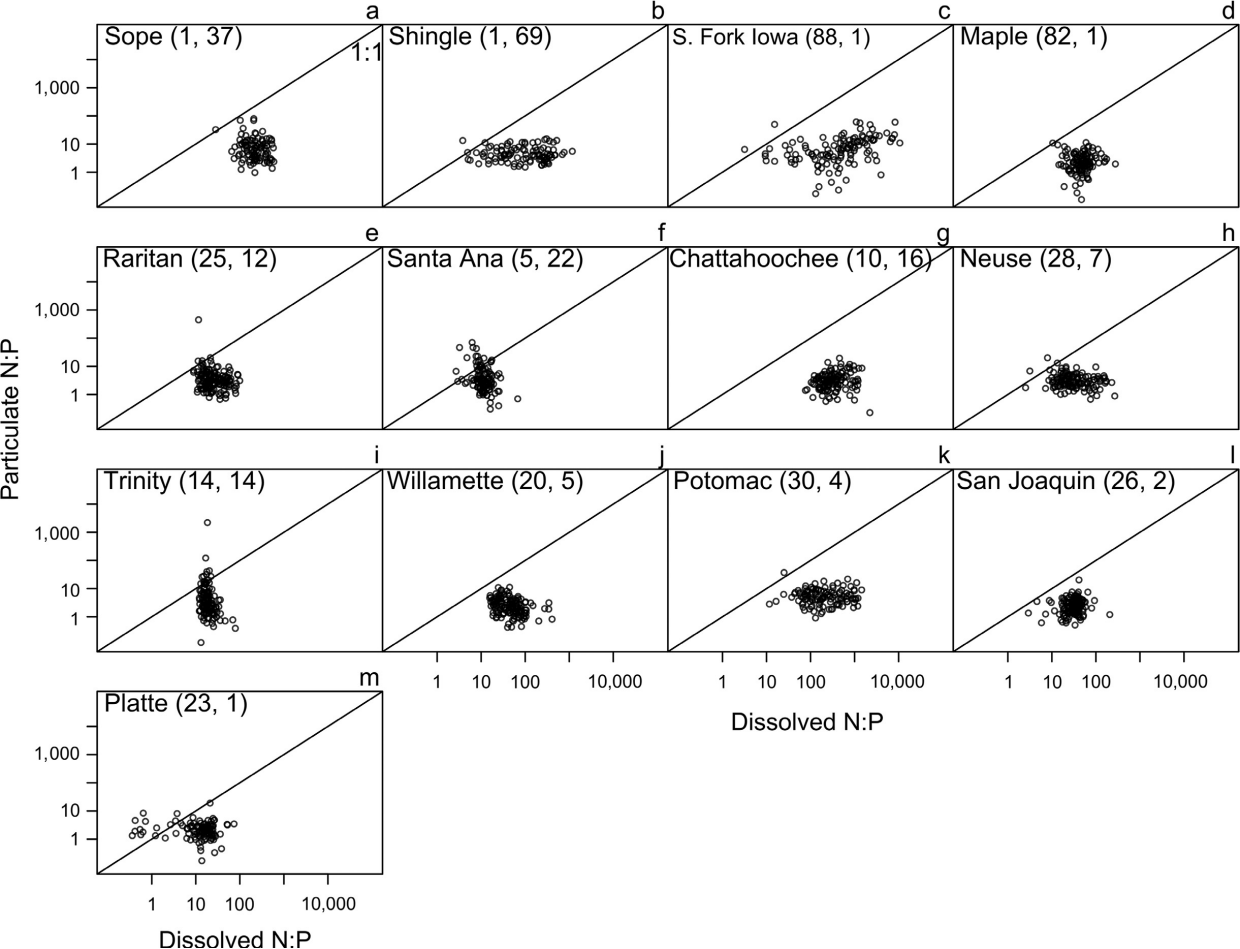
Particulate N:P as a function of dissolved N:P in the subset of 13 streams that had >100 sequential samples. Distinct patterns emerging between the two nutrient forms among rivers suggest that the relationship between dissolved and particulate N:P in rivers may be a quantifiable characteristic of a stream or river that gives insight into its function (e.g., nutrient transport, primary production). Numbers in parentheses after each stream or river name indicate corresponding % agriculture and % urban land cover, respectively. Note that both *x‐* and *y*‐axes are presented on log_10_ scale. All ratios are molar.

Consistent with the pattern observed for all streams (Fig. 6), 7 of 13 streams had weak or no relationship between particulate N:P ratios and dissolved N:P ratios, suggesting static particulate N:P ratios (Fig. 8b, d, g, h, k, l, m; Table 2). However, we found evidence for a relationship between particulate N:P ratios and dissolved N:P ratios in some individual streams (i.e., 1/*H* ≠ 0; Table 2, Fig. 8). One stream (South Fork Iowa River) showed a weak positive relationship (Fig. 8c, Table 2). Five of 13 streams showed negative trends, indicating lower particulate N:P ratios with high DIN:DIP (Table 2); some negative trends occurred when DIN:DIP variability was relatively constrained (e.g., Fig. 8i; Trinity River, TX, USA).

## Discussion

Our findings underscore that both N and P have reached concentrations in U.S. streams and rivers that are above documented thresholds for altered stream ecosystem structure and functioning, including primary production, macroinvertebrate and fish communities, and organic matter decomposition (Dodds et al. 2002, Evans‐White et al. 2013, Kominoski et al. 2015, Schmidt et al. 2019). U.S. states are currently developing stressor‐response‐based nutrient criteria for streams and rivers (Evans‐White et al. 2013). The elevated concentrations of nutrients reported here are indicative of conditions associated with ecological impairment across the U.S., and while elevated concentrations have been previously documented (Alexander and Smith 2006, Herlihy and Sifneos 2008, Stoddard et al. 2016), the spatial and temporal patterns of N and P concentrations, and their effects, warrant increased attention and evaluation to avoid future degradation of stream ecosystems (e.g., Stets et al. 2020). With the caveat that the NAWQA sampling design may disproportionately represent sites with greater human impacts under baseflow conditions, we nonetheless report N and P concentrations in excess of ecosystem structure and function thresholds that occurred at low levels of agricultural or urban development (between 5–15% of the watershed). Even in these watersheds with relatively minor agriculture or urbanization, 26% of TN concentrations exceeded 1.162 mg/L and 33% of TP concentrations exceeded 0.074 mg/L, the most conservative thresholds for benthic algae responses reported by Evans‐White et al. (2013). This suggests that lower land use cover by agricultural and urban uses cannot be used as a proxy for negligible nutrient inputs and suggests monitoring and best management practices across diverse landscapes are needed. Further, 32% of values reported here exceeded the highest ecoregional reference condition for TN (2.5 mg/L), and 30% of values exceeded the highest ecoregional reference condition for TP (0.181 mg/L; Herlihy and Sifneos 2008), indicating the magnitude of the nutrient pollution problem in the U.S. currently.

Our analysis of N:P ratios in U.S. streams and rivers also supports the idea that nutrient enrichment has shifted N:P ratios to higher values, especially for dissolved fractions. In both agricultural and urban watersheds, dissolved N concentrations were especially elevated, whereas P concentrations were relatively greater in particles, consistent with previous studies (Meybeck 1982, Uusitalo et al. 2003, Scott et al. 2007). These patterns point to the potential for greater transport of dissolved N relative to P to downstream ecosystems, while P is transported relatively more than N in suspended particles. Control of both N and P are critical to reducing excess nutrients in U.S. streams and rivers, and strategies to control their concentrations should consider how differing human‐influenced sources affect the stoichiometry of N and P as they cycle between dissolved and particulate forms.

### Watershed land‐use, hydrology, and stream N:P ratios

Different land‐use practices and atmospheric inputs affect N:P ratio signatures in lakes (Arbuckle and Downing 2001, Elser et al. 2009), but few studies have fully defined how pervasive nutrient loading from diverse watershed sources is manifested in terms of nutrient availability in streams, which may be at least as sensitive to human inputs (Maranger et al. 2018). Contrary to the hypothesis that urbanization may increase P more than N (Duan et al. 2012, McDowell et al. 2019), we found both agriculture and urbanization increased dissolved and total N concentrations, suggesting that watersheds with intensive human activities are generally characterized by greater N loading relative to P. However, increases in N tended to be weaker in urban vs. agricultural watersheds. We also found that particulate P concentrations tended to be higher with greater urbanization, suggesting a stronger signal of particle‐derived P in these watersheds (e.g., possibly from diffuse sources such as increased soil or streambank erosion; [Withers and Jarvie 2008, Fox et al. 2016] and/or particles influenced by treated or untreated sewage or septage). This pattern, coupled with visual trends (e.g., Fig. 4), indicated that urban watersheds are characterized by lower N:P relative to agricultural watersheds (also see Hobbie et al. 2017).

Stream flow rate affected stream N:P ratios, especially in the case of total N:P in agricultural watersheds. Site‐specific discharge‐N:P ratio relationships were largely indicative of changes to N:P ratios with higher flows; as discharge increases, the N:P ratios of dissolved nutrients increase while those of particulate N:P ratios decrease, whereas we found roughly equivalent evidence for increases and decreases of total N:P ratios with increasing discharge. These findings are consistent with previously documented trends indicating relatively more N is transported in dissolved forms, and relatively more P is transported in particulates (e.g., Uusitalo et al. 2003, Scott et al. 2007) Agricultural watersheds tended to show greater prevalence for positive discharge‐N:P ratio slopes, consistent with the pattern of greater N availability with higher agricultural land use. Increased frequency of high‐flow events may exacerbate high N:P in these watersheds. In highly urban watersheds (i.e., > 50% urban), the relationships between dissolved N:P ratios and discharge appeared to weaken (approaching zero), indicating that N and P sources and transport are relatively consistent across flow conditions in watersheds affected by urbanization (Fig. 5).

### Dissolved and particulate N:P ratios

Clear differences emerged between dissolved and particulate N:P across U.S. streams and rivers. Dissolved N:P tended to be more variable and showed higher N:P ratios overall. Particulate N:P spanned a much smaller range of values and was more P‐rich. Considering stream nutrient fractions in terms of dissolved N:P supply and N:P in particles supported the hypothesis that particulate N:P is relatively constant across dissolved N:P availability. This pattern is in stark contrast to oceans, where there is stronger congruence between dissolved N:P and particulate N:P (Sterner and Elser 2002). Although our use of a stoichiometric model implies a biological mechanism for this pattern, abiotic drivers could be equally or more important. For instance, agricultural watersheds (both row‐crop and pasture) may be especially prone to highly dynamic and elevated transport of dissolved N and P (especially N for row‐crops), while increased sediment erosion contributes to relatively lower, and consistent N:P of particles (Table 1). This finding also indicates that transported particles exhibit N:P ratios that are lower than seston and microbial biomass in marine, lake, and terrestrial ecosystems (Copin‐Montegut and Copin‐Montegut 1983, Hecky et al. 1993, Cleveland and Liptzin 2007, Sterner et al. 2008). Given that particulate forms of N and P are more likely to be retained, and exhibit greater retention times than dissolved fractions (Newbold et al. 1983, Hall et al. 2009), stoichiometric constancy of particulate N:P could result in enhanced delivery of dissolved N to downstream ecosystems, and relatively greater retention of particulate P.

Another unresolved question is the fate of transported particulate P upon reaching downstream estuaries and coastal oceans. Based on our findings, we may speculate that the greater oxygenation of streams and rivers compared to oceans likely favors precipitation of P with iron (Fe) contributing to greater transport of P in particles. However, in estuaries and coastal oceans with less oxygen, more sulfur (S), and less Fe, the P that was in particles as iron oxyhydroxides could become available in river deltas and coastal oceans (e.g., Blomqvist et al. 2004). This potential effect combined with enhanced delivery of dissolved N also emphasizes the importance of targeted, dual reductions of dissolved N and particulate P delivery to coastal regions.

The remarkably constant particulate N:P ratios we observed likely imply a common source of these nutrients across the watersheds that we analyzed, but pinpointing the potential sources remains a difficult task. For example, in an analysis of soil N:P stoichiometry, Cleveland and Liptzin (2007) observed that overall N:P of bulk soils was 13:1, a value higher than the central tendencies of particulate N:P reported here that suggests that upland soils do not dominate suspended particulate fractions in streams and rivers. Despite the differences between previously reported soil N:P and stream particle N:P presented here, the well‐known hydrologic connectivity between streams and their adjacent landscapes (Dodds and Whiles 2004), suggests that stream particles reflect more proximal sources such as streambank erosion (Fox et al. 2016). Analyses of stream, river, and reservoir sediment N:P ratios suggest that the suspended particulate matter N:P reported here is essentially equivalent to that of river sediments (geometric mean N:P suspended particles = 3.98 [this study], geometric mean N:P river sediment = 3.1 [Sinsabaugh et al. 2012]), and buried sediments in reservoirs (N:P = 4–6, Vanni et al. 2011). This result adds weight to the evidence that stream particles, whether settled or suspended, reflect greater P content than do soil particles. We are unable to directly link riparian or upland nutrient sources to the composition of stream particles during downstream transformation and transport using this data set, but this analysis points to the need for further exploration of particle nutrient sources that could reveal the drivers of similar nutrient stoichiometry among riparian soils, suspended particles, and benthic sediments.

### Temporal patterns in dissolved and particulate N:P

The subset of 13 streams with decadal‐scale nutrient concentration data revealed similar patterns to those we observed in the larger spatial data set: dissolved N:P was variable, while particulate N:P was relatively constant in all cases. Underlying mechanisms for trends in riverine dissolved N:P should be explored further (e.g., contemporary or legacy sources of P [Stackpoole et al. 2019], denitrification, reduced N loads, etc.). Seven streams had 1/*H* values close to zero, indicating N:P stasis and consistent with the larger data set. However, some streams showed some noteworthy anomalies. For instance, the South Fork Iowa River (Iowa, USA) exhibited a weak positive relationship between particulate N:P and dissolved N:P, and approached what could be considered stoichiometric plasticity. The South Fork Iowa River may be susceptible to such a pattern given that it contained very high agricultural land use (>80% in the watershed) and therefore particles in this stream may reflect prevalence of stoichiometrically flexible phytoplankton or microbial biomass in the water column. Future analyses of stream functions could consider the potential for a signal between dissolved vs. particulate nutrients and ecosystem functions such as primary production or whole‐stream metabolism.

Larger rivers, such as the Raritan, Willamette, Potomac, and Platte, appeared to have periodic fluctuations in dissolved N:P without similar changes to particulate N:P (see Appendix S1: Figs. S3, S4). For example, in the Raritan River, these fluctuations were characterized by higher DIN:DIP during winter (mean DIN:DIP = 55:1 [December‐February]) and lower DIN:DIP during summer (often approaching Redfield N:P; mean DIN:DIP = 18:1 [June–August]). However, some larger rivers showed relatively constant DIN:DIP ratios and highly variable particulate N:P ratios (e.g., Trinity River, TX, USA); these rivers sometimes exhibited negative relationships between DIN:DIP and particulate N:P, suggesting that when DIN:DIP is high, particulate N:P is low. The mechanisms driving these negative relationships remain unclear; high‐flow events may play a role in re‐suspending P‐rich particles and temporarily increasing dissolved N concentrations.

### Implications for ecosystem function and dual nutrient management in streams and rivers

In the context of N and P concentrations and their potential effects on ecosystem structure and functions, the streams and rivers analyzed here frequently exhibited N and P concentrations that exceed previously reported thresholds at which biological structure and processes are modified and/or stimulated (e.g., half‐saturation constants, thresholds, or stressor‐response relationships; Dodds et al. 2002, Evans‐White et al. 2013, Kominoski et al. 2015, Fig. 1). Median N and P concentrations exceeded values that are predicted to increase stream benthic algal biomass and stimulate breakdown of organic matter (Dodds et al. 2002, Woodward et al. 2012, Fig. 1). For example, shifts in stream benthic algal community structure have been documented when streamwater TP exceeds 21 μg/L (Taylor et al. 2014), and benthic algal biomass has been shown to increase when TN and TP exceed 40 and 30 μg/L, respectively (Dodds et al. 2002); median TN and TP concentrations reported here are ~ 36× and 3 × these respective thresholds. Leaf litter breakdown is also stimulated at very low N and P concentrations, ranging from ~ 16–52 μg/L dissolved N (Ferreira et al. 2006, Kominoski et al. 2015) and ~ 5–21 μg/L dissolved P (Rosemond et al. 2002, Ferreira et al. 2006, Kominoski et al. 2015). Primary and secondary consumer communities can also exhibit threshold responses to increasing nutrient concentrations. The median TN and TP concentrations we report are 1.35 and 1.81 × higher than reported thresholds for changes to macroinvertebrate communities (Evans‐White et al. 2009), and 3.23 × higher than a reported TP threshold for fish communities (Taylor et al. 2014). We note that the thresholds we used were conservative, representing the highest values reported in the literature. These thresholds are not absolute, and structural and functional responses have been documented at lower concentrations (Evans‐White et al. 2013). In addition, the magnitude of biological responses to nutrients can scale with the degree of enrichment (Ferreira et al. 2015). From this standpoint, our results, combined with the co‐limitation of ecosystem processes by N and P (Elser et al. 2007, Ferreira et al. 2015), the scaling of biological responses to concentrations of N and P (Ferreira et al. 2015), and associated concentration‐dependent responses to both nutrients (Kominoski et al. 2015), all suggest that control of both N and P is required to avoid proliferation of algae, accelerated loss rates of detritus in streams, and changes to stream consumer communities.

Nutrient pollution in U.S. streams and rivers remains a persistent and complex problem, exacerbated by the diffuse sources of nutrients across watersheds – many linked to multiple human activities. Our analysis emphasizes the key role that humans play in the nutrient regimes of streams and rivers, and stresses how even relatively minor human activity can increase nutrient concentrations in freshwaters, with the most pronounced effects for dissolved N compared to P, resulting in higher N:P ratios across the coterminous U.S. Indeed, the majority of streams analyzed here had N:P ratios that exceeded Redfield N:P of 16:1, implying that reduction of excessive N inputs to streams should be prioritized over P at large spatial scales. However, the relatively high concentrations of N underscores the importance of N removal for controlling eutrophication downstream, where N is more likely to limit primary production (e.g., in an estuary). Relatively high N concentrations also likely exacerbate P limitation of stream biota, and therefore they might be more responsive to management practices that reduce P concentrations. Further, what remains unclear is how much excessive N concentrations are solely dependent on inputs rather than the degree to which severe P limitation impedes N removal processes (Finlay et al. 2013), thereby intensifying N accumulation. In addition, while a focus on minimizing N inputs could be recommended, we also found evidence for coupled relationships between increasing N and P concentrations, especially for total and particulate nutrients in urban streams. This pattern is suggestive of common sources for both nutrients in these watersheds and so efforts to reduce total N inputs could also potentially reduce P inputs and concentrations. Next steps within management frameworks to curb nutrient concentrations in streams and rivers should consider relationships between N and P, as well as other limiting elements (e.g., Si; McDowell et al. 2019) especially over decadal, high‐frequency time‐series, to understand short‐term, seasonal and long‐term changes to nutrient inputs and internal processing.

## Supporting information

Appendix S1

## Data Availability

Associated R code and the specific data used for this analysis are available on Zenodo: https://doi.org/10.5281/zenodo.3688886
