# Supplementary material for: Transport of N and P in U.S. streams and rivers differs with land use and between dissolved and particulate forms
Source: Ecol Appl. 2020 May 5;30(6):e02130. doi: 10.1002/eap.2130 (PMC7507146; doi:10.1002/eap.2130)
Supplement: Supplementary file 1 — Appendix S1 [file EAP-30-e02130-s001.pdf]

Supporting Information. Manning, D. W. P., A. D. Rosemond, J. P. Benstead, P. M. Bumpers, and J. S. Kominoski. 2020. Transport of N and P in U.S. streams and rivers differs with land use and between dissolved and particulate forms. Ecological Applications.

Appendix S1.

Table S1. Parameter codes (http://nwis.waterdata.usgs.gov/usa/nwis/pmcodes), their descriptions, and standard methodologies used for the nutrient data obtained for this analysis. Methods for each analyte were obtained from the National Environmental Methods Index (https://www.nemi.gov/home/).

Parameter Code	Description	Method description	Method ID
Nitrogen			
00608	Ammonia, water, filtered, milligrams per liter as nitrogen	colorimetry	I-2252-90
00631	Nitrate plus nitrite, water, filtered, milligrams per liter as nitrogen	cadmium reduction-diazotization/colorimetry	I2545-90
62855	Total nitrogen [nitrate + nitrite + ammonia + organic-N], water, unfiltered, analytically determined, milligrams per liter	alkaline persulfate digest	I4650-03
62854	Total nitrogen [nitrate + nitrite + ammonia + organic-N], water, filtered, analytically determined, milligrams per liter		I4650-03
Phosphorus			
00665	Phosphorus, water, unfiltered, milligrams per liter as phosphorus	acid-persulfate digest/ molybdenum blue method	I-4607-90
00666	Phosphorus, water, filtered, milligrams per liter as phosphorus	acid-persulfate digest/ molybdenum blue method	I-2607-90
00671	Orthophosphate, water, filtered, milligrams per liter as phosphorus	molybdenum blue method	I-2601-90

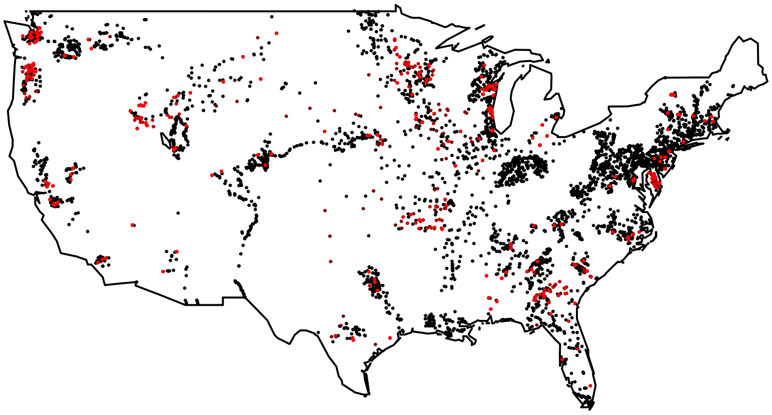

Figure S1. Map of the all sites included in the USGS NAWQA study (*n* = 2070; black circles), and the remaining sites that contained corresponding dissolved and total N and P values (*n* = 439, red circles). Thirty-eight (38) states were represented in the smaller data set analyzed here (states not represented in the smaller data set were AK, HI, IN, KY, LA, ME, MS, ND, NH, NM, RI, VT).

Supplemental Methods: Accounting for left-censoring in the data set

The NAWQA data set contained values that were listed as “less than” a given value, typically the analytical detection limit (e.g., 0.001 mg/L). We conducted our analysis with and without these values to ascertain their effects on the inferences we were able to draw from these data. We also used Monte Carlo simulations to generate estimates of NH_4-N, NO_3-N, and dissolved inorganic phosphorus (DIP) when these were listed as “less than” a certain value in the data set. In general, issues with reported detection limits were more common with dissolved forms of N and P vs. total N and P, which only had 4 and 38 values listed as “less than”, respectively, out of 7,954 values. Therefore, we focused our sensitivity analysis on the dissolved forms of N and P.

There was uneven representation of values below limits of detection that depended on the nutrient concentration type. Ammonium-N (NH_4-N) data contained the greatest percentage of “less than” values (n = 3,280, or ~41% of the final data set we used); however, NH_4-N rarely comprised more than 10% of dissolved inorganic nitrogen concentrations (~25% of cases), and was below 4% of dissolved inorganic nitrogen concentrations in 50% of cases. In contrast, NO_3-N and DIP had fewer instances where “less than” values were an issue (n = 407 or 5.1% of the data for NO_3-N, n = 561, or 9.1% of the data for DIP). The number of values reported as “estimated” was comparable to those reported as “less than” (% “estimated” = 2.3, 14.3, and 9.2% for NO_3-N, NH_4-N, and DIP).

We used Monte Carlo simulations to model DIN and DIP values that were reported as “less than” a given value (reported limits of detection: NO_3-N = 0.016-0.06; NH_4-N = 0.01-0.2; DIP = 0.004-0.024). We replaced any value that was reported as “less than” with the median value of 1000 random samples from a uniform distribution that ranged from 0 to the reported

detection limit (e.g., $U \sim [0, \text{detection limit}]$). To model values listed as “estimated” we replaced any value with this designation with 1000 random samples from a normal distribution with mean = ith “estimated” value, and an analyte-specific standard deviation based on the reported precision of the corresponding analytical method (see Table S1). Replacing the DIN or DIP values in these two ways (hereafter, ‘modeled’) had negligible effects on the distribution of DIN:DIP ratios (Kolmogorov-Smirnov test; $D = 0.018$; $P = 0.123$). Specifically, the median DIN:DIP ratio computed from the data replaced with simulated values was 55, while the median DIN:DIP ratio of the unmodified data was 54. The range of values was comparable, with DIN:DIP ratios calculated from simulated data ranging from 0.16 to 11,994, and unmodified DIN:DIP ratios ranging from 0.15 to 15,177. Mean modeled DIN:DIP ratios were higher than unmodified DIN:DIP ratios (181 vs. 163, respectively). Nevertheless, we used modeled DIN:DIP ratios in our analysis to ensure that we accounted for uncertainty in the DIN or DIP estimates that were reported as “less than” or “estimated” in the data set.

Supplemental Data and Methods: Total dissolved vs. particulate N and P estimates

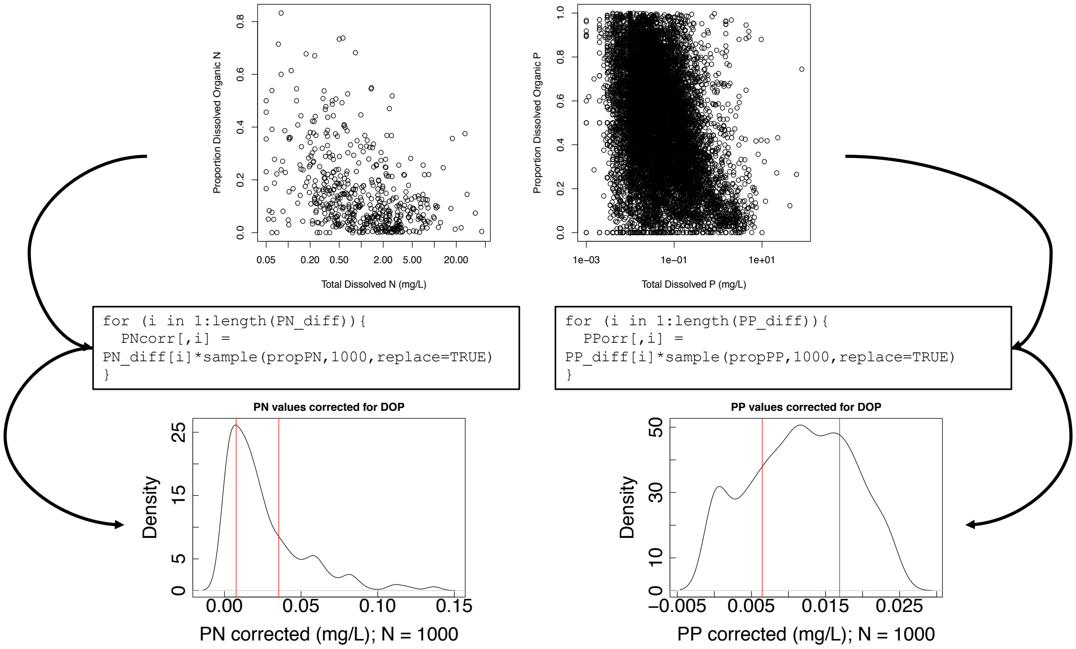

Fig. S2. Plots illustrating the relationship between estimated proportions of dissolved organic nitrogen (DON) or dissolved organic phosphorus (DOP) and total dissolved N and P (TDN, TDP, respectively) and example workflows for bootstrap resampling procedures. The proportion of DON and DOP were determined by calculating the difference between total N and P and total dissolved N and P (total dissolved nitrogen [TDN] and total dissolved phosphorus [TDP]). Data were retrieved using the '*dataRetrieval*' package in R that retrieves data made available by USGS and EPA (Hirsch and DeCicco 2015). Linear regression indicated that the proportion of DON or DOP in a sample was related to ln-transformed total dissolved nutrient concentrations (prop. DON = -0.043*ln(TDN) + 0.15, $P < 0.001$; $R^2 = 0.14$; prop. DOP = -0.26*ln(TDP) +

0.501; P = <0.001, R^2 = 0.049). The percentage of particulate N (% PN) out of total N, decreased as a function of agricultural land use (% PN = -0.204* *% agriculture* + 20.78; P = 0.0297), but was unrelated to urban land use. The percentage of particulate P (% PP) out of total P was unrelated to % agriculture, and % urban land use. We accounted for uncertainty in our particulate N and P data using bootstrap resampling (as described in the main text). We iteratively multiplied the *i*th particulate N and P estimate (i.e., estimated by subtraction of undigested DIN and DIP values from TN and TP) with 1000 random samples from distributions of the DON and DOP proportions. The proportion DON and DOP were approximately normally distributed with mean (±1 standard deviation) of 0.15 (0.15) and 0.48 (0.27), respectively. We then used a random value from within the interquartile range (e.g., red vertical lines in the two example density plots) of the resulting 1000 simulated DON and DOP corrections for the estimated particulate N and P in our analyses.

References

Hirsch, R. M., and L. A. De Cicco. 2015. User guide to exploration and graphics for RivEr Trends (EGRET) and dataRetrieval: R packages for hydrologic data (version 2.0, February 2015): U.S. Geological Survey Techniques and Methods book 4, chap. A10, 93 p., http://dx.doi.org/10.3133/tm4A10

Table S2. Summaries of nutrient concentration data across the U.S. in watersheds with agricultural and urban land use (% agriculture + urban). We report 10th, 25th, 50th, 75th and 90th percentile values for total, dissolved, and particulate nutrients (TN, TP, DIN, DIP, PN, PP) across watersheds with differing levels of % agriculture + urban. All concentrations are in mg/L. Agriculture + urban land use at the watershed level were combined, and binned with a 10% window around the 10th, 25th, 50th, 75th and 90th percentiles of this land use metric.

5-15% agriculture + urban

Percentile	TN	DIN	PN	TP	DIP	PP
10%	0.222	0.049	0.009	0.012	0.003	0.004
25%	0.315	0.096	0.015	0.022	0.004	0.007
50%	0.548	0.229	0.026	0.051	0.016	0.012
75%	1.165	0.742	0.045	0.105	0.037	0.024
90%	2.115	1.646	0.073	0.198	0.094	0.045

20-30% agriculture + urban

Percentile	TN	DIN	PN	TP	DIP	PP
10%	0.492	0.148	0.013	0.036	0.007	0.008
25%	0.725	0.351	0.022	0.054	0.016	0.014
50%	1.632	1.011	0.041	0.096	0.041	0.026
75%	3.080	2.402	0.075	0.398	0.192	0.055
90%	5.556	4.625	0.140	0.990	0.728	0.119

45-55% agriculture + urban

Percentile	TN	DIN	PN	TP	DIP	PP
10%	0.747	0.182	0.018	0.025	0.004	0.007

25%	0.981	0.432	0.028	0.041	0.009	0.013
50%	1.262	0.744	0.049	0.087	0.025	0.023
75%	1.581	1.181	0.076	0.138	0.051	0.041
90%	2.592	1.930	0.121	0.198	0.098	0.062

70-80% agriculture + urban

Percentile	TN	DIN	PN	TP	DIP	PP
10%	0.916	0.303	0.013	0.043	0.007	0.007
25%	1.293	0.684	0.027	0.066	0.019	0.015
50%	2.134	1.575	0.055	0.120	0.055	0.030
75%	4.604	4.062	0.096	0.242	0.132	0.049
90%	7.362	6.631	0.145	0.735	0.546	0.083

85-95% *agriculture + urban*

Percentile	TN	DIN	PN	TP	DIP	PP
10%	1.145	0.649	0.020	0.051	0.013	0.010
25%	1.839	1.189	0.038	0.086	0.033	0.019
50%	4.965	3.685	0.071	0.188	0.088	0.037
75%	10.231	9.232	0.142	0.346	0.187	0.080
90%	15.623	14.026	0.281	0.941	0.406	0.210

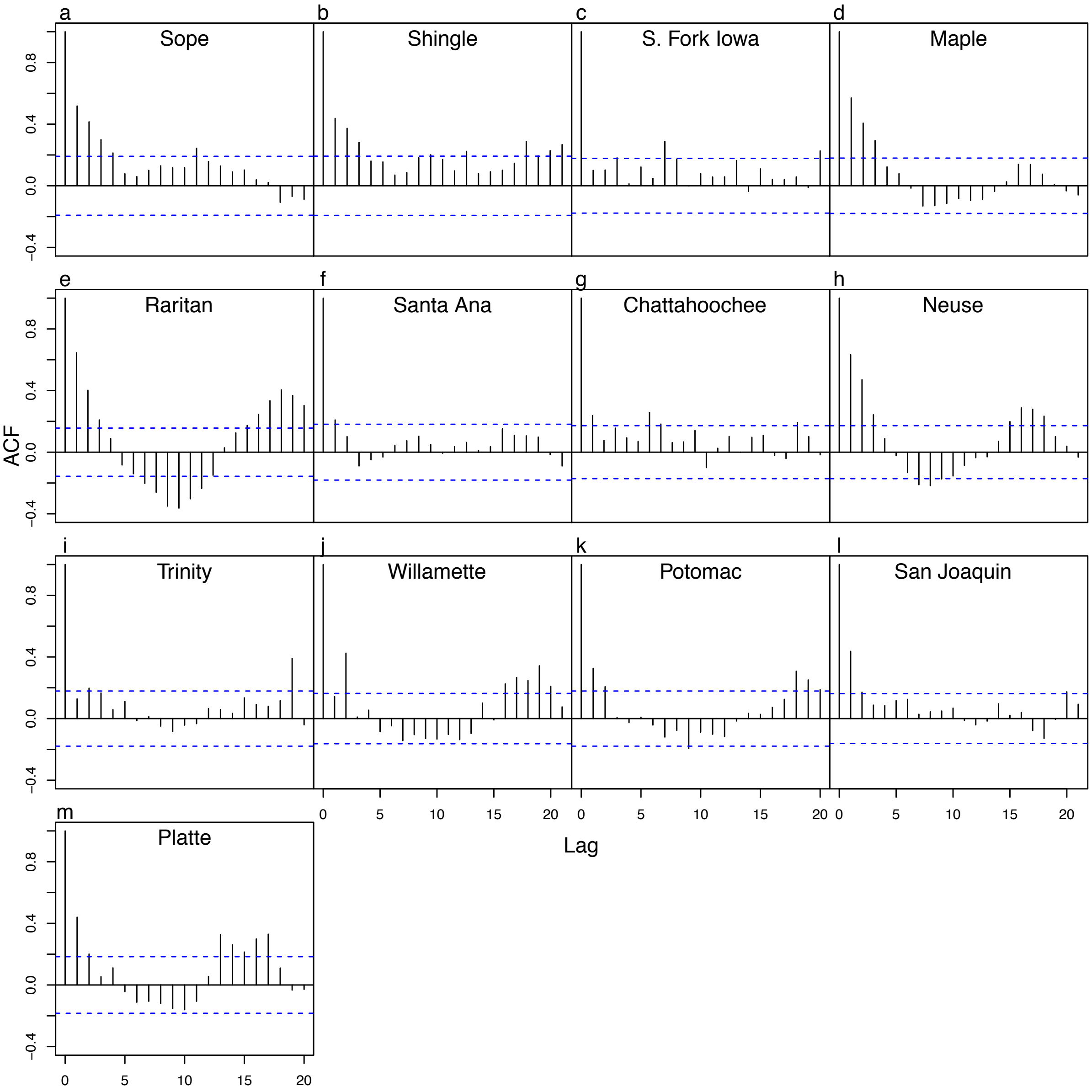

Figure S3. Autocorrelation functions for the time-series of DIN:DIP ratios for the 13 streams or rivers with more than 100 samples. Horizontal blue dashed lines indicate values above or below which significant correlations exist for a specific time-lag (e.g., lag 10 = ten time intervals; streams or rivers were sampled ~monthly).

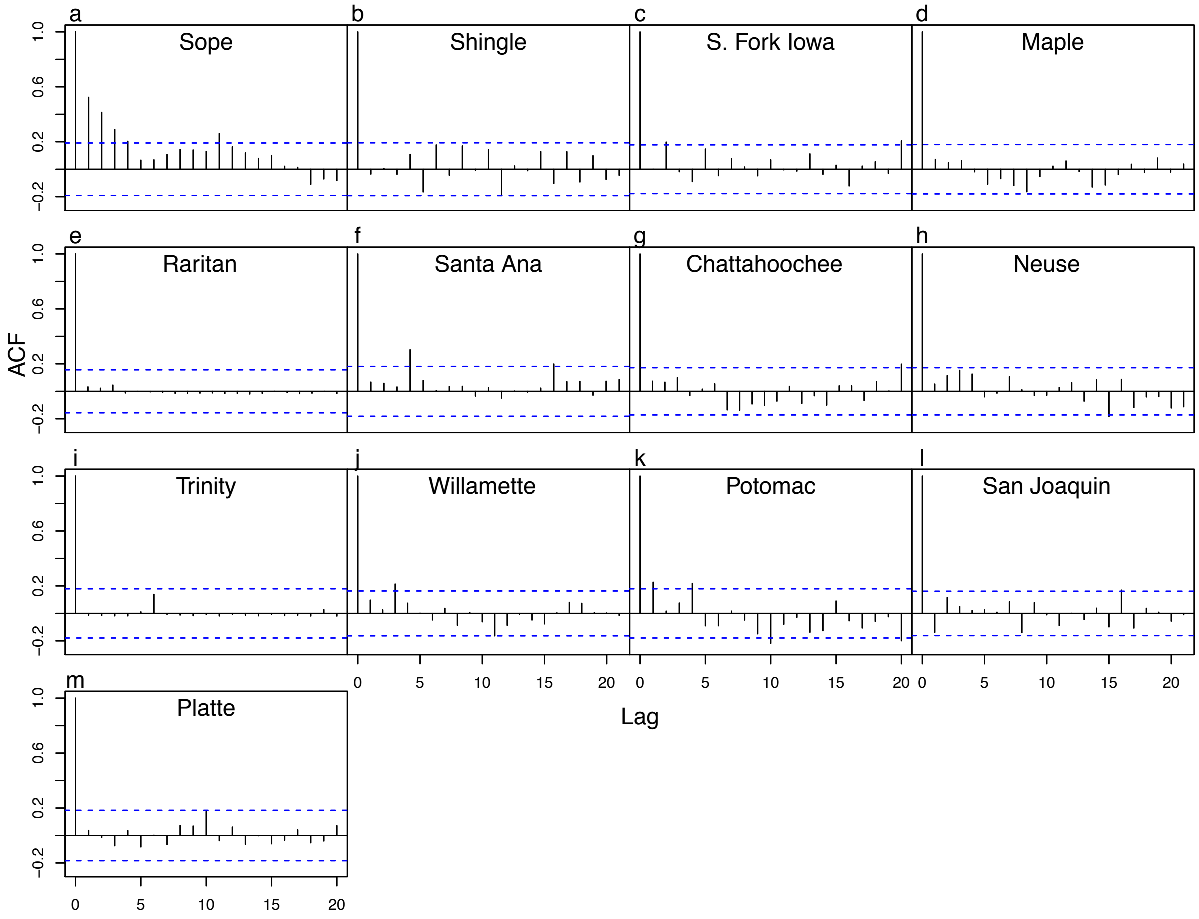

Figure S4. Autocorrelation functions for the time-series of particulate N:P ratios for the 13 streams or rivers with more than 100 samples. Horizontal blue dashed lines indicate values above or below which significant correlations exist for a specific time-lag (e.g., lag 10 = ten sampling-intervals; streams or rivers were sampled ~monthly).
